# CRISPR‐Cas13d effectively targets SARS‐CoV‐2 variants, including Delta and Omicron, and inhibits viral infection

**DOI:** 10.1002/mco2.208

**Published:** 2023-01-31

**Authors:** Zongzhi Liu, Xiang Gao, Chuanwen Kan, Lingyu Li, Yuan Zhang, Yibo Gao, Shengyuan Zhang, Liangji Zhou, Hui Zhao, Mingkun Li, Zheng Zhang, Yingli Sun

**Affiliations:** ^1^ Central Laboratory National Cancer Center/National Clinical Research Center for Cancer/Cancer Hospital and Shenzhen Hospital Chinese Academy of Medical Sciences and Peking Union Medical College Shenzhen China; ^2^ University of Chinese Academy of Sciences Beijing China; ^3^ CAS Key Laboratory of Genome Sciences and Information Beijing Institute of Genomics Chinese Academy of Sciences/China National Center for Bioinformation Beijing China; ^4^ Institute of Hepatology National Clinical Research Center for Infectious Disease School of Medicine Shenzhen Third People's Hospital The Second Affiliated Hospital Southern University of Science and Technology Shenzhen Guangdong China; ^5^ Beijing Institute of Genomics Chinese Academy of Sciences, China National Center for Bioinformation Beijing China; ^6^ Key Laboratory for Regenerative Medicine Ministry of Education School of Biomedical Sciences Faculty of Medicine The Chinese University of Hong Kong Hong Kong; ^7^ National Cancer Center/National Clinical Research Center for Cancer/Cancer Hospital Chinese Academy of Medical Sciences and Peking Union Medical College Beijing China; ^8^ Kunming Institute of Zoology, The Chinese University of Hong Kong (KIZ‐CUHK) Joint Laboratory of Bioresources and Molecular Research of Common Diseases The Chinese University of Hong Kong Hong Kong; ^9^ Hong Kong Branch of CAS Center for Excellence in Animal Evolution and Genetics The Chinese University of Hong Kong Hong Kong; ^10^ Key Laboratory of Genomic and Precision Medicine Beijing Institute of Genomics Chinese Academy of Sciences China National Center for Bioinformation Beijing China

**Keywords:** CRISPR‐Cas system, gene editing, RNAi therapy, SARS‐CoV‐2

## Abstract

The recent pandemic of variants of concern (VOC) of severe acute respiratory syndrome coronavirus 2 (SARS‐CoV‐2) highlights the need for innovative anti‐SARS‐CoV‐2 approaches in addition to vaccines and antiviral therapeutics. Here, we demonstrate that a CRISPR‐Cas13‐based strategy against SARS‐CoV‐2 can effectively degrade viral RNA. First, we conducted a cytological infection experiment, screened CRISPR‐associated RNAs (crRNAs) targeting conserved regions of viruses, and used an in vitro system to validate functional crRNAs. Reprogrammed Cas13d effectors targeting NSP13, NSP14, and nucleocapsid transcripts achieved >99% silencing efficiency in human cells which are infected with coronavirus 2, including the emerging variants in the last 2 years, B.1, B.1.1.7 (Alpha), D614G B.1.351 (Beta), and B.1.617 (Delta). Furthermore, we conducted bioinformatics data analysis. We collected the sequence information of COVID‐19 and its variants from China, and phylogenetic analysis revealed that these crRNA oligos could target almost 100% of the SARS‐CoV family, including the emerging new variant, Omicron. The reprogrammed Cas13d exhibited high specificity, efficiency, and rapid deployment properties; therefore, it is promising for antiviral drug development. This system could possibly be used to protect against unexpected SARS‐CoV‐2 variants carrying multiple mutations.

## Graphical abstract

- Cas13d‐crRNAs inhibit both ancestral and mutated SARS‐CoV‐2 replication.
- Cas13d‐crRNAs inhibit both ancestral and mutated SARS‐CoV‐2 replication including Delta.
- Cas13d‐crRNAs could inhibit Omicron and other SARS family strains and are a potential pan‐SARS inhibition strategy.

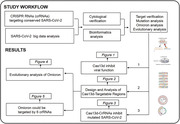

## 1 INTRODUCTION

Severe acute respiratory syndrome coronavirus 2 (SARS‐CoV‐2) has caused >647 million infections and over 6.6 million deaths worldwide as of December 2022 (PAHO Weekly COVID‐19 Epidemiological Update), it remains an open question regarding the origin, the transmission capacity, and the immune‐escape potential of the variant.^1^, ^2^ The rapid mutagenesis of SARS‐CoV‐2 in both the environment and the human body is demonstrated by rapidly emerging variants of concern (VOC). Effective vaccines,^3^ monoclonal antibodies,^4^, ^5^ and antiviral drugs^6^, ^7^ are facing challenges of new VOCs in eliminating the SARS‐CoV‐2 pandemic because of the rapid mutagenesis of the RNA genome of SARS‐CoV‐2.

SARS‐CoV‐2, as an RNA virus, easily undergoes genetic evolution with new host or time, resulting in the emergence of many different variants. In less than 1 year, three SARS‐CoV‐2 VOC, B.1.1.7, B.1.351, and P.1 (referred to as Alpha, Beta, and Gamma, respectively), rapidly developed into predominant sources of infections.^8^, ^9^, ^10^, ^11^, ^12^, ^13^, ^14^ Recently, new variants, including B.1.427 (Epsilon), B.1.526 (Iota), and B.1.617 (Delta) have emerged, and variant B.1.617 has become the greatest concern because it has a high infection rate and spread quickly in many countries.^15^ More recently, Omicron (B1.1.529) caused the majority of new infections worldwide. Both Delta and Omicron could escape the current vaccines for SARS‐CoV‐2. Therefore, it raises the serious concern that the rapid mutagenesis and “immune escape” of new SARS‐CoV‐2 VOCs could compromise our efforts toward controlling the pandemic.^16^ Therefore, there is an urgent need for effective approaches to treat and eliminate SARS‐CoV‐2.

CRISPR‐Cas13 is an adaptive immunity form that existed in bacteria, and it can suppress bacteriophage RNA.^17^, ^18^, ^19^ Previous work showed that Cas13 can degrade genomic RNAs of viruses in mammalian cells.^20^, ^21^, ^22^ However, whether Cas13 can recognize and degrade RNAs of SARS‐CoV‐2 viruses and mutated variants such as Delta and Omicron and even new unknown variants remains to be illustrated.

The novel coronavirus causing COVID‐19 is an RNA virus.^23^ SARS‐CoV‐2 binds to angiotensin‐converting enzyme 2 (ACE2) receptor and invades the cells, releases its RNA into the cells, and generates mRNAs in the replication cycle.^24^, ^25^ Most ongoing vaccine trials work with antibodies or chemical drugs that inhibit protein function.^26^ We performed an alternative antiviral approach based on a CRISPR13d‐based system to eliminate RNAs of SARS‐CoV‐2 and its fast spreading variants, which are developed much more faster than antibodies, drugs, and vaccines.

To inhibit SARS‐CoV‐2 and its variants, strategies using Cas13a have been proposed.^27^ Considering the size, efficiency, and delivery, we used the recently reported CRISPR‐Cas13d system, an RNA‐guided RNA endonuclease,^28^, ^29^ which is much more efficient than Cas13a. Cas13d can bind to CRISPR‐associated RNAs (crRNAs), which carry a well‐designed 22‐nt spacer sequence. The crRNAs can direct the Cas13d enzyme to specific targeted RNA sequences for degradation. The Cas13d RNA endonuclease provides a powerful tool for degradation of SARS‐CoV‐2 RNAs. With specificity, efficiency, and small size (967 amino acids), Cas13d overperformed other Cas13 families to target and destroy RNAs.^29^, ^30^, ^31^ Although this is not the first study to apply Cas13d to target viral RNA sequences.^20^, ^32^ However, to the best of our knowledge, there is no evidence to prove whether CRISPR‐Cas13d can be designed to target and cleave authentic SARS‐CoV‐2 and its variants, including Delta and Omicron, in infected human cells. Our approach is novel because it developed a strategy for simultaneous targeting of different strains of authentic SARS‐CoV‐2, including new emerging strains with unexpected mutations, such as Delta and Omicron.

**FIGURE 1:**
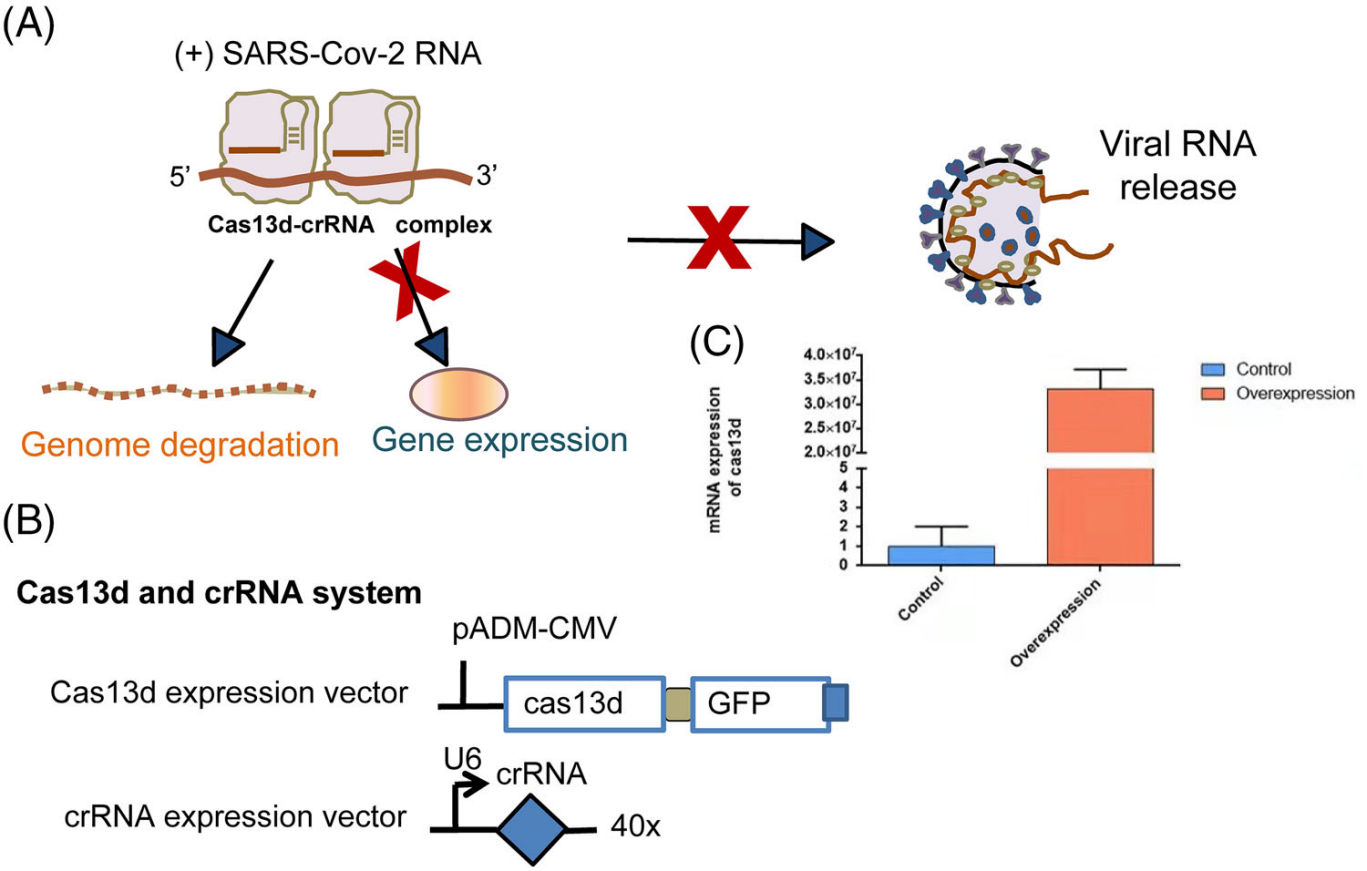
The hypothetical inhibition of severe acute respiratory syndrome coronavirus 2 (SARS‐CoV‐2) RNA and replication by reprogrammed Cas13d. (A) Cas13d can inhibit viral function and replication by directly targeting and cleaving all viral positive‐sense RNA. (B) Schematics of the constructs used to express Cas13d or CRISPR‐associated RNAs (crRNAs). (C) Reprogrammed Cas13d was dramatically over‐expressed in HeLa‐angiotensin‐converting enzyme 2 (ACE2) cells

In this paper, we developed an antiviral CRISPR Cas13d system as a genetic intervention targeting SARS‐CoV‐2 (Figure 1), unexpected mutants of SARS‐CoV‐2, and potentially all strains of SARS family, and SARS‐CoV‐2 variants. We used bioinformatic tools to search for highly conserved regions across all existing SARS‐CoV‐2 genomes and targeted these regions for viral in vitro and in vivo degradation. We tested our strategy first with synthetic fragments of SARS‐CoV‐2, and then with live infected cells using four SARS‐CoV‐2 variant strains in human cells. We designed crRNA pools and screened crRNAs targeting the conserved viral regions of SARS‐CoV‐2 variants and defined six crRNAs (Table S1) with the highest efficiency. We proved that our Cas13d strategy can degrade SARS‐CoV‐2 fragments and inhibit the infection of authentic SARS‐CoV‐2 variants in human cells. Our bioinformatics analysis showed that the six crRNAs could also target unexpected mutations, such as Omicron, with only one nucleotide mismatch in six crRNAs. Therefore, we developed a reprogrammed CRISPR‐psp Cas13d complex that can efficiently degrade SARS‐CoV‐2, including that with the recently emerging dominant VOC, B.1, B.1.1.7 (Alpha), D614G B.1.351 (Beta), B.1.617 (Delta), and the unexpected mutants of SARS‐CoV‐2, such as Omicron. This strategy provides a powerful and rapid tool to protect against RNA virus infections in the future.

## 2 RESULTS

### 2.1 Design of crRNAs and bioinformatic analysis of Cas13d target regions in the virus genome

**FIGURE 2:**
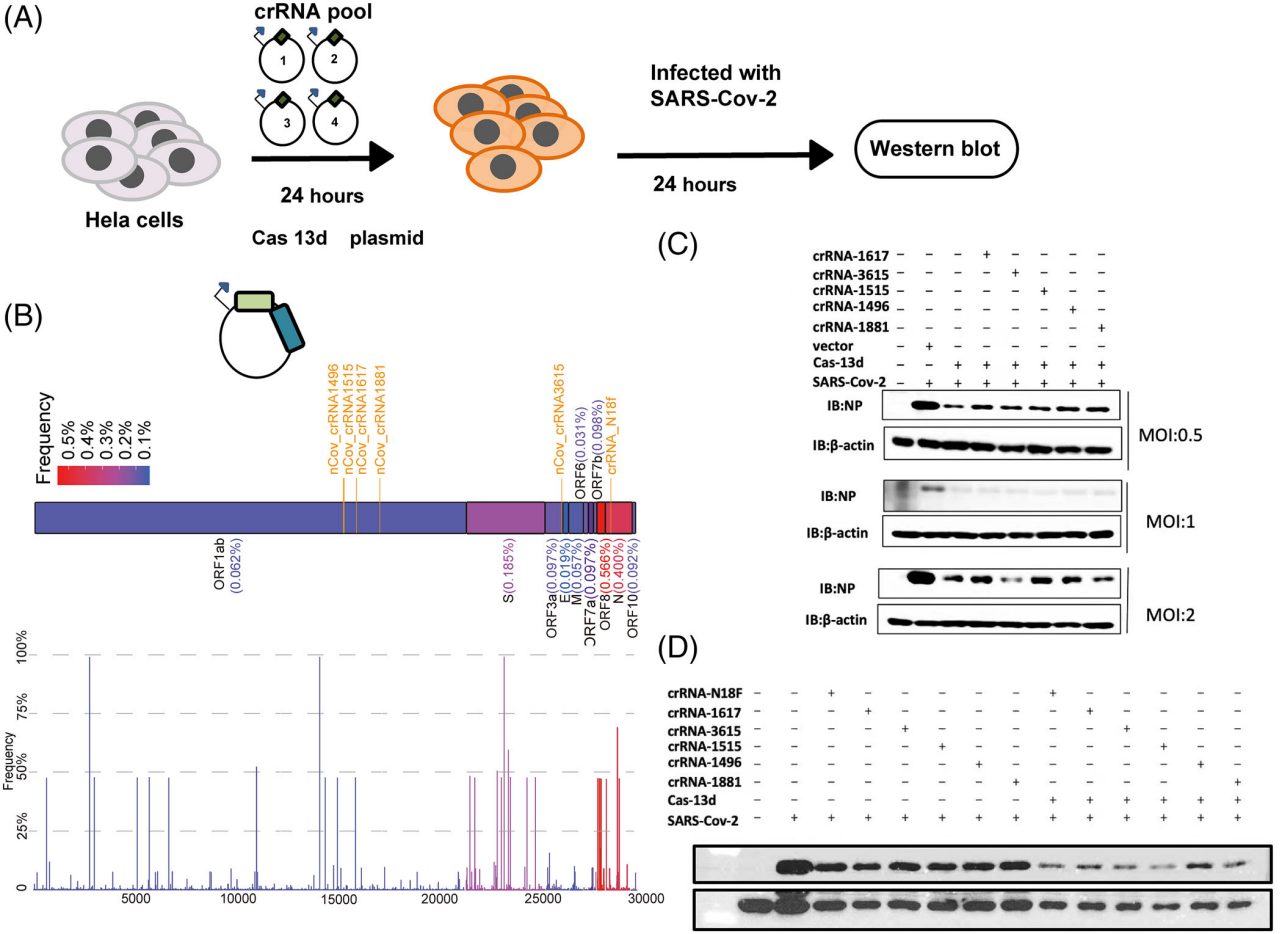
Computational design and analysis of Cas13d‐targetable regions in the severe acute respiratory syndrome coronavirus 2 (SARS‐CoV‐2) genome, schematics of the transfection and inhibition of SARS‐CoV‐2 in human cells, and western blot showing inhibition capability of different CRISPR‐associated RNAs (crRNAs) on SARS‐CoV‐2 Wuhan strain. (A) Experimental workflow of transfection and inhibition of SARS‐CoV‐2 RNA replication in human HACE cells by programmed Cas13d and crRNAs. (B) Frequency of mutated bases. The number of mutations of the SARS‐COV‐2 sequence in the corresponding protein region in the database was calculated and divided by the length of the corresponding protein region as the mutation frequency of the protein region (indicated in different colors according to the size of the mutation frequency), and the positions of the six crRNAs in the protein region are indicated in orange. The bar chart below shows the mutation frequency of each site in different protein regions, with different colors distinguishing different protein regions. (C) The Cas13d and crRNAs system suppressed different doses of ancestral SARS‐CoV‐2 infection. HeLa‐angiotensin‐converting enzyme 2 (ACE2) cells were transfected with plasmids encoding crRNAs and Cas13d for 24 h then infected with ancestral SARS‐CoV‐2 (SZTH‐003) at 0.5 multiplicity of infection (MOI),1 MOI, and 2 MOI for 24 h at 37°C. The cell lysates were analyzed by western blotting. (D) The Cas13d and crRNA system suppressed the ancestral SARS‐CoV‐2 infected HeLa‐ACE2 cells. HeLa‐ACE2 cells were transfected with plasmids encoding crRNAs and Cas13d for 24 h and then infected with ancestral SARS‐CoV‐2 (SZTH‐003) at 1 MOI for 24 h at 37°C. The cell lysates were analyzed by western blotting

We used a bioinformatics analysis aligning published SARS‐CoV‐2 genomes from NCBI to develop crRNA sequences to target SARS‐CoV‐2. The SARS‐CoV‐2 virus genome contains 3000 bp RNA strand that encodes 12 functional open reading frames.^33^, ^34^ Our analysis figured out regions that are highly conserved among all the SARS‐CoV‐2 strains available (Figure 2 and Table S2). Two of the most highly conserved regions in the ORF1ab region were chosen, NSP13 and NSP14, which maintain the important function in all SARS‐CoV‐2 strains, and the nucleocapsid (*N*) gene.

We collected all possible crRNA sequences and analyzed these sequences to design crRNAs. crRNAs that are predicted to have potential off‐target binding (≤2 mismatches) or possess poly‐T (≥4 Ts) sequences that may prevent crRNA expression were excluded. Then, we used an in vitro system to screen for the most effective crRNAs, and we obtained a collection of 50 crRNAs (Table S3),^35^ with 20 crRNAs targeting the *OFR1ab* and *N* genes. An in vitro assay showed that targeting these two regions could dramatically reduce RNA fragments of SARS‐CoV‐2 required for virus replication.

### 2.2 Screening of crRNAs for inhibiting the expression of SARS‐CoV‐2 fragments

To test whether the Cas13d we generated was effective, we used an in vitro system with synthetic fragments of F1 and F2. To test the in vivo efficiency of Cas13d cleavage for authentic viruses, we used HeLa‐ACE2 cells for infection and inhibition. We expressed Cas13d and crRNA through adenovirus infection and detected Cas13d overexpression using RT‐PCR. The cell‐free system was prepared, as previously described. The SARS‐CoV‐2 fragment was added to the 20 μl cell‐free system, and RT‐PCR was performed to detect the degradation of the fragments.

Quantitative real‐time PCR (qRT‐PCR) analysis showed that most crRNA pools could degrade the fragments compared to that in the control. crRNA‐N18 and crRNA‐3615, targeting the conserved region of the SARS‐CoV‐2‐N fragment, were particularly effective, and they degraded the fragments by over 99% (Table S4). crRNAs that target the *ORF1ab* genes crRNA‐1496, crRNA‐1515, crRNA‐1617, and crRNA‐1881 substantially degraded SARS‐CoV‐2‐ORF1ab, causing an over 99% decrease (Table S4). The ability of different crRNAs to degrade virus RNA could be explained by the RNA secondary structure, which causes different binding affinities of each crRNA sequence.

Cas13d could be an effective system for targeting and degrading SARS‐CoV‐2 RNAs in human cells. Careful design of crRNAs is critical to achieve efficient SARS‐CoV‐2 inhibition, and thousands of crRNAs could be screened with an in vitro system using virus fragments before validation using authentic viruses in P3 laboratory.

### 2.3 Cas13d‐crRNA inhibits authentic SARS‐CoV‐2 infection in human cells

A genetically engineered H1N1 influenza virus was used to detect the activity of Cas13d.^36^ Another study evaluated Cas13b and not Cas13d^27^; however, Cas13d is much less effective in degrading RNA. To test whether our Cas13 generated could inhibit authentic SARS‐CoV‐2 RNA in human cells, we transfected HeLa‐ACE2 cells with Cas13d and the six crRNAs, which are the most effective in vitro systems in human cell models (Figure 2). HeLa‐ACE2 cells were plated into 24‐well plates and transfected 24 h later with 1 μg crRNA and Cas13d, 24 h following which they were infected with authentic SARS‐CoV‐2(614D) at a multiplicity of infection (MOI) of 0.3 (Figure 2A,B). We optimized the transfection conditions of HeLa‐ACE2 cells to achieve 70%–90% transfection efficiency based on GFP expression and RT‐PCR detection in HeLa‐ACE2 cells. Post‐transfection (after 24 h), cells were infected with authentic SARS‐CoV‐2 at an MOI of 0.5–2. We assayed the culture medium at 24 h using western blotting for assessing the NP levels.^37^ The viral load was reduced by the reduction in the coronaviral NP levels, as shown (Figure 2C). The crRNA and Cas13d system could inhibit high rates (2 MOI) of SARS‐CoV‐2 infection (Figure 2C).

**FIGURE 3:**
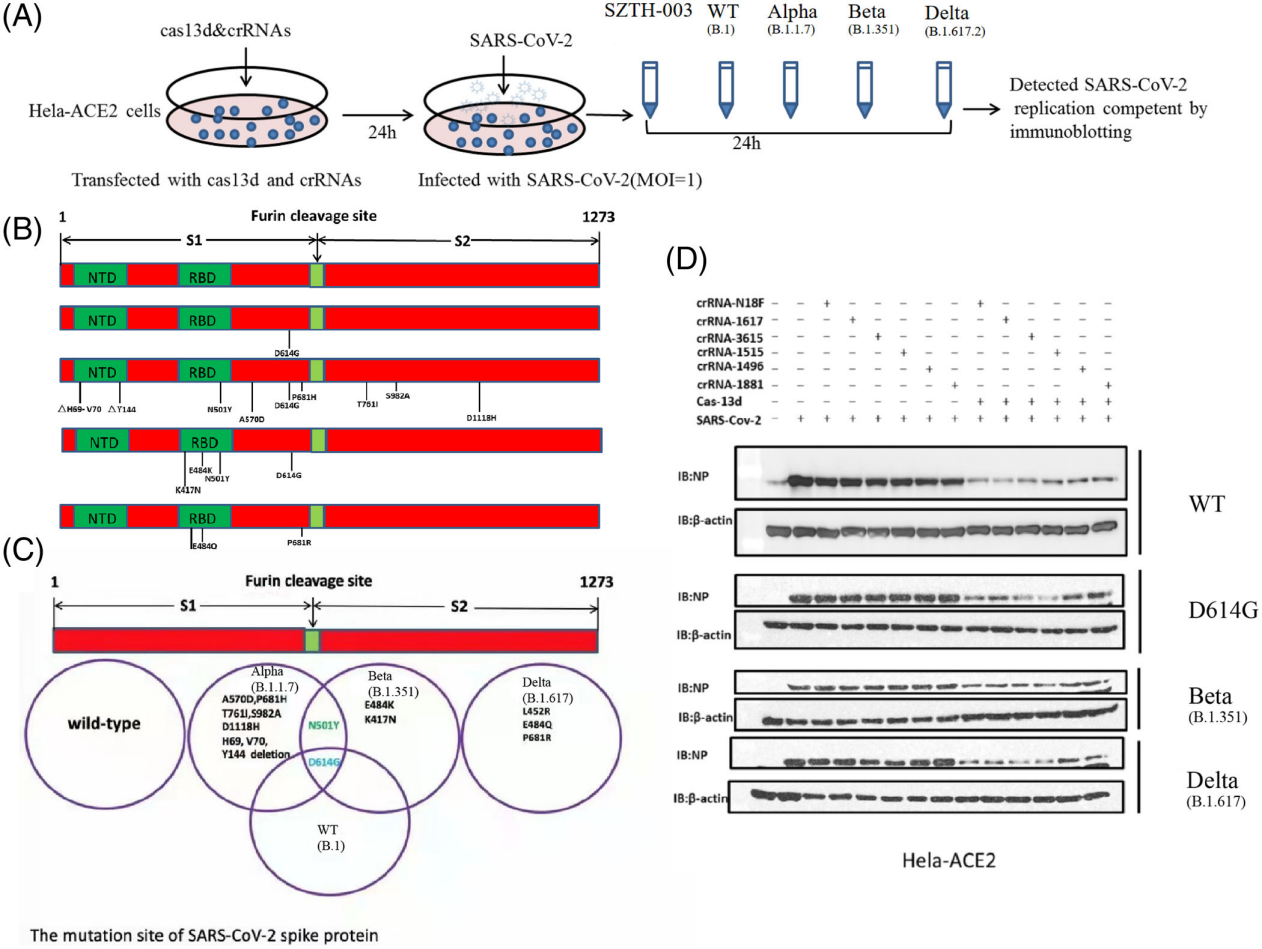
Cas13d‐CRISPR‐associated RNAs (crRNAs) inhibit both ancestral and mutated severe acute respiratory syndrome coronavirus 2 (SARS‐CoV‐2) replication, including Delta, in vivo. (A) Schematic diagram of Cas13d‐mediated suppression of SARS‐CoV‐2 in infected HeLa‐angiotensin‐converting enzyme 2 (ACE2) cells. HeLa‐ACE2 cells were transfected with Cas13d and various crRNAs for 24 h and then infected with ancestral (614D, SZTH‐003), WT (B.1), Alpha (B.1.1.7), Beta (B.1.351), and Delta (B.1.617) SARS‐CoV‐2 for 24 h. The level of NP was detected by western blotting. (B) Schematic representation of the location of mutated site domain arrangement of the SARS‐CoV‐2 spike protein. B.1: SARS‐CoV‐2 spike protein substitution (D614G); Alpha (B.1.1.7): there are two sites with deletions (positions 69–70, 144–145) and seven sites with amino acid substitutions (N501Y, A570D, D614G, P681H, T761I, S982A); Beta (B.1.351) (K417N, E484K, N501Y, D614G); Delta (B.1.617) (L452R, E484Q, D614G, P681R). (C) Schematic representation of the location of mutated site domain arrangement of the SARS‐CoV‐2 spike protein and comparison of different SARS‐CoV‐2 mutants causing the current pandemic. (D) The Cas13d and crRNA system could suppress emerging SARS‐CoV‐2 mutant strain infection. HeLa‐ACE2 cells were transfected with plasmids encoding crRNAs and Cas13 and infected with four SARS‐CoV‐2 strains for 24 h. The cell lysates were analyzed by western blotting using indicated antibody. NTD: N‐terminal domain; RBD: receptor binding domain

Targeting different regions of SARS‐CoV‐2 viral RNA with a mix of different crRNAs would increase the efficiency of degradation and inhibition. We transfected HeLa‐ACE2 cells with a pool of crRNAs containing six different crRNAs targeting either the SARS‐CoV‐2 structural protein NCP or the non‐structural proteins NSP13 and NSP14.^7^, ^38^, ^39^ We infected the HeLa‐ACE2 cells with SARS‐CoV‐2 and quantified the viral protein in the culture supernatant. All crRNAs significantly reduced viral protein levels and replication (Figure 3).

### 2.4 Cas13d‐crRNAs inhibit both ancestral and mutated SARS‐CoV‐2 replication in vivo

**FIGURE 4:**
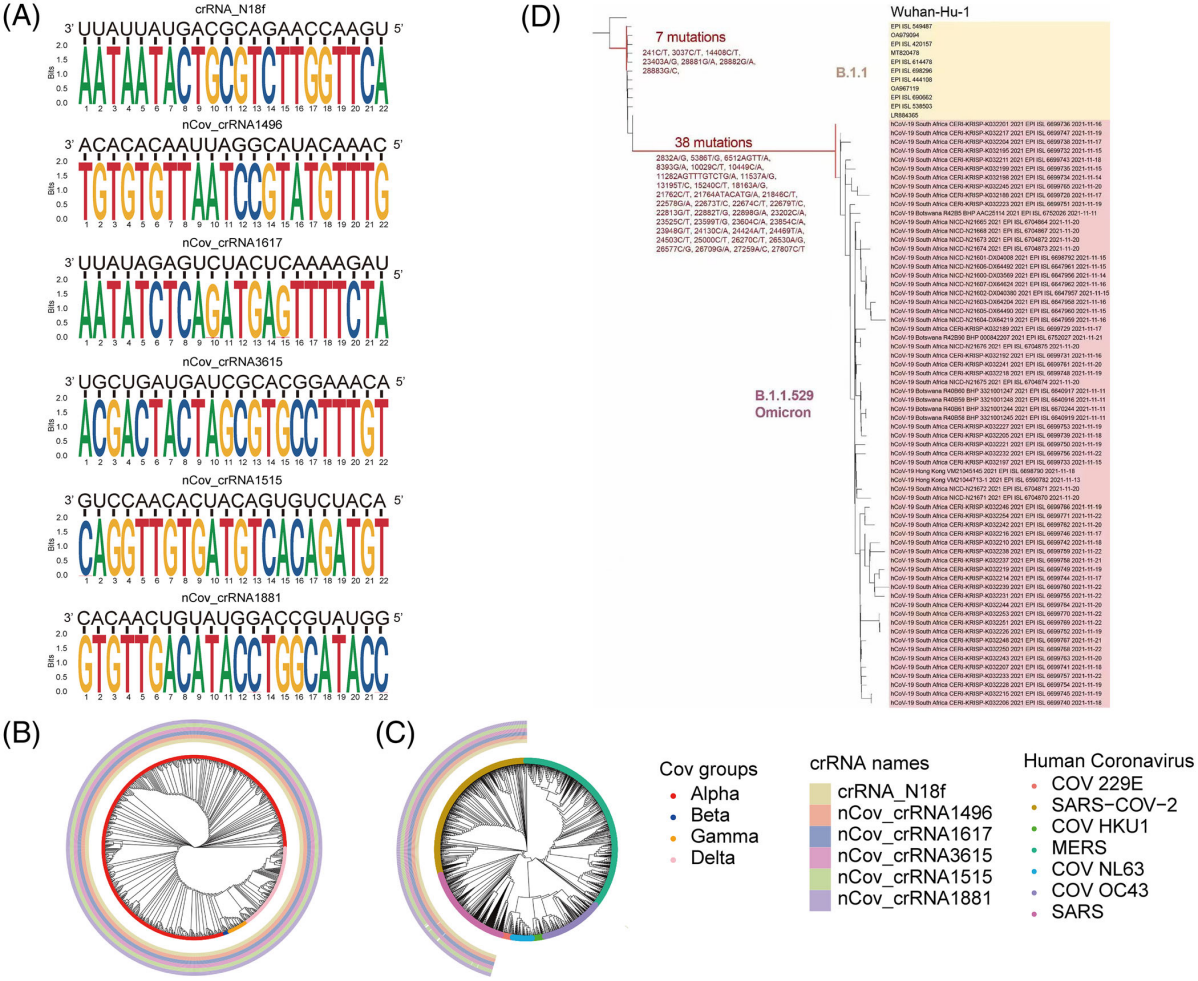
Sequence analysis of CRISPR‐associated RNA (crRNA) targeting efficiency in severe acute respiratory syndrome coronavirus 2 (SARS‐CoV‐2) family and evolutionary analysis of the sequence of Omicron. (A) The logo of six different crRNAs that showed the highest inhibition efficiency locating on the most conserved regions of SARS‐CoV‐2. (B) The six crRNAs can target Alpha, Beta, Gamma, and Delta strains. The type of SARS‐CoV‐2 strains to which the sequence belongs and whether crRNA can target the sequence (less than or equal to two mutations occur in the complementary pairing region of crRNA) are annotated on the evolutionary tree. (C) Among all human coronaviruses, the six crRNAs can only specifically target the SARS family. The type of coronaviruses to which the sequence belongs and whether crRNA can target the sequence (less than or equal to two mutations occur in the complementary pairing region of crRNA) are annotated on the evolutionary tree. (D) Evolutionary tree of Omicron showing that it contains more mutations than its relatives

**FIGURE 5:**
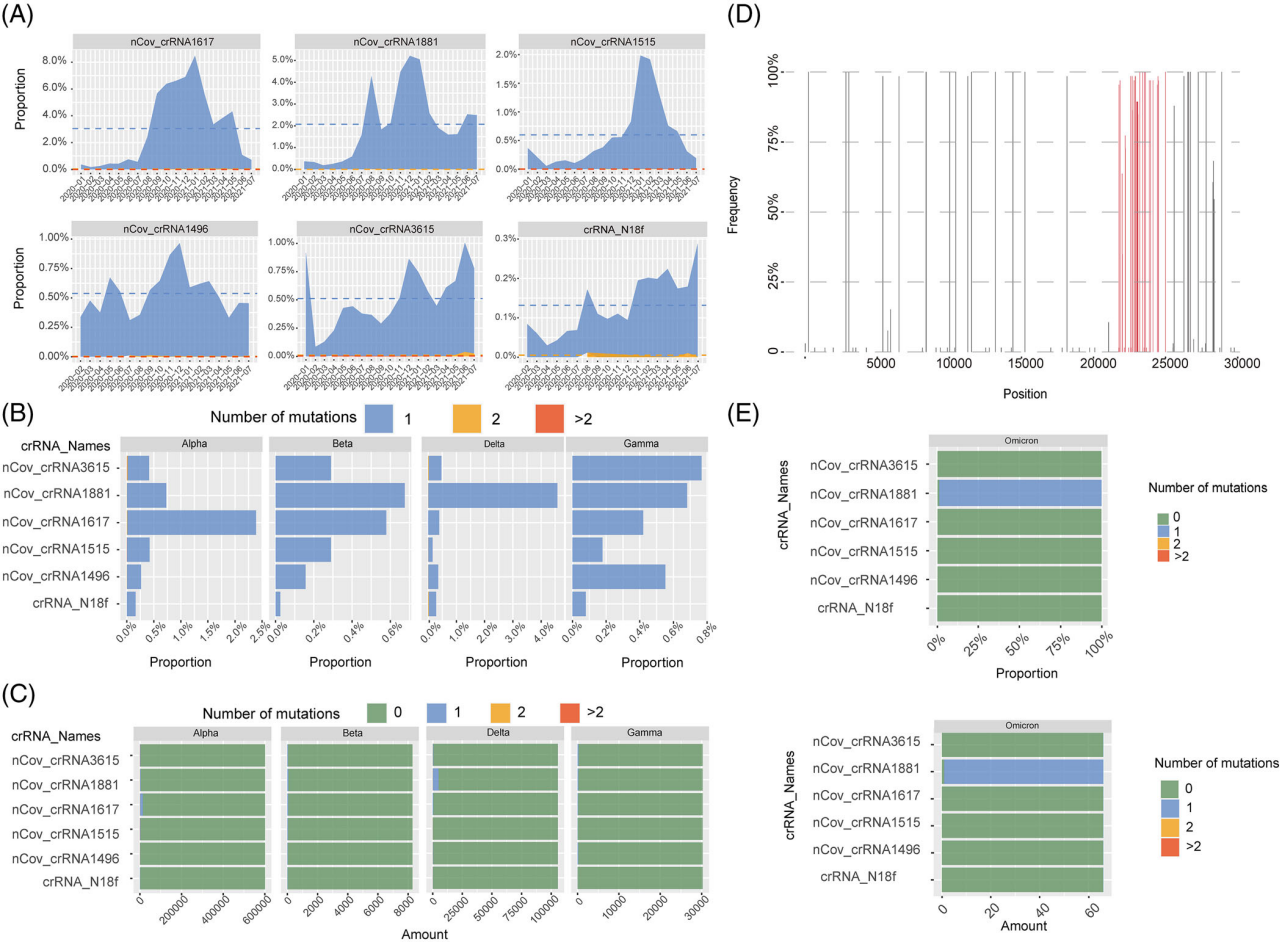
Data analysis showed that the six CRISPR‐associated RNAs (crRNAs) target conserved regions of severe acute respiratory syndrome coronavirus 2 (SARS‐CoV‐2), and all current mutations could not affect its specificity and mismatches are very rare. Sequence analysis also predicted that Omicron could be targeted by these six crRNAs. (A) The proportion of the number of mutations in the six crRNA sequence alignment regions of SARS‐CoV‐2 sequences changed over time. (B) The proportion of the number of mutations in the six crRNA sequence alignment regions of SARS‐CoV‐2 sequences belonging to four different novel coronavirus variants. (C) The number of mutations in the crRNA sequence alignment regions of SARS‐CoV‐2. (D) The mutation distribution of Omicron strain. The horizontal axis shows the location of the SARS‐CoV‐2 sequence, and the vertical axis reflects the proportion of mutations in omicron strains at different locations. The region highlighted in red is the sequence region that encodes the S protein. (E) The proportion of mutations in the six crRNA sequence alignment regions of SARS‐CoV‐2 sequences belonging to Omicron. For crRNA_N18f, nCov_crRNA1496, nCov_crRNA1515, nCov_crRNA1617, and nCov_crRNA3615 sequences, 100% of SARS‐CoV‐2 sequence had 0 mutation sites. For nCov_crRNA1881 sequence, 1.52% of the SARS‐CoV‐2 sequence had 0 mutation site, 98.48% had 1 mutation site. Two mutation sites and more than two mutations accounted for 0. (F) The number of mutations in the six crRNA sequence alignment regions of SARS‐CoV‐2 sequences belonging to Omicron. For crRNA_N18f, nCov_crRNA1496, nCov_crRNA1515, nCov_crRNA1617, and nCov_crRNA3615 sequences, 66 sequences had 0 mutation site. For nCov_crRNA1881 sequence, one SARS‐CoV‐2 sequence had 0 mutation site, and 65 sequences had one mutation site

SARS‐CoV‐2 variants are currently pandemic, which enables continuous mutation of their genomes. This puts tremendous pressure on epidemic prevention and control, especially on the development of vaccines. We determined whether the crRNA and Cas13d system could suppress the emerging SARS‐CoV‐2 epidemic strains (including mutant strains of B.1, B.1.351, B.1.1.7, and B.1.617). Our data showed that the Cas13d and crRNA system could suppress all SARS‐CoV‐2 mutant strains (Figures 4 and 5A–C,E). Cas13d and crRNA degraded the RNA of viruses, as indicated by the decrease in coronaviral NP levels. The inhibition of B.1.351 was marginally more effective than that of the other three SARS‐CoV‐2 variants. Overall, this study provides strong evidence that the system of crRNA and Cas13d efficiently silences both the ancestral and emerging SARS‐CoV‐2 strains.

### 2.5 Cas13d‐crRNAs could inhibit Omicron and other SARS family strains and is a potential pan‐SARS inhibition strategy

The Omicron mutant contains 26–32 mutations in the S protein, which are in the ACE2 receptor‐binding domain. These mutations are possibly responsible for escape from vaccines or neutralizing antibodies.^40^ Bioinformatics analysis showed six crRNAs that could also target unexpected mutations, such as Omicron, with only one nucleotide mismatch in the six crRNAs (Figure 5D–F).

Many variants of SARS are highly infectious, causing pandemic and high death rate.^41^, ^42^, ^43^ Therefore, novel method that could broadly target and prevent unexpected virus threats from SARS is urgently needed. Here, we use a minimal number of crRNAs to target existing and unexpected SARS strains, and it is of great value to apply this strategy with worldwide spread of mutations and variants.

We analyzed a SARS sequence available to find crRNAs and finally screened out six crRNAs. The high efficacy of a small number of crRNAs to effectively target most or all variants highlights the unique advantage of this approach. We have included a map to show how the top six crRNAs target the SARS phylogenetic tree (Figure 4C and Table S5). Middle East respiratory syndrome (MERS) was not covered by these six crRNAs.

A recent study analyzed over a million SARS‐CoV‐2 genomes and found that different subtypes (L and S) of SARS‐CoV‐2^44^ can be covered by these six crRNAs (Figure 5A–C and Table S6). crRNA‐1496 targets 1,651,447 out of 1,651,448 (99.9999%) SARS‐CoV‐2 sequences. crRNA‐1515 targets 1,651,443 out of 1,651,448 (99.9997%) SARS‐CoV‐2 sequences. crRNA‐1617 targets 1,651,444 out of 1,651,448 (99.9998%) SARS‐CoV‐2 sequences. crRNA‐1881 targets 1,651,448 out of 1,651,448 (100.0%) SARS‐CoV‐2 sequences. crRNA‐3615 targets 1,651,444 out of 1,651,448 (99.9998%) SARS‐CoV‐2 sequences. crRNA‐N18 targets 1,651,448 out of 1,651,448 (100.0%) SARS‐CoV‐2 sequences. These results showed that our approach can efficiently target different variants and can be easily applied for unexpected mutations of VOCs. These bioinformatic analyses were also proved by our in vivo work using authentic SARS‐CoV‐2 viruses.

## 3 DISCUSSION

In recent years, many variants of SARS‐CoV‐2 have caused pandemic and a significantly high rate of fatalities.^41^, ^42^, ^43^ These variants include Alpha (B.1.1.7), Beta (B.1.351), Gamma (P.1), and Delta (B.1.617.2), which were first found in the United Kingdom, South Africa, Brazil, and India. Omicron is also a VOC mutant (https://www.who.int/activities/tracking‐SARS‐CoV‐2‐variants/). Although all kinds of SARS‐CoV‐2 vaccines and monoclonal antibodies are available or under development, they are threatened due to the rapid mutation and immune escape of new variants, including Delta and Omicron. Therefore, a fast and easy strategy that could catch up with emergence of unexpected SARS‐CoV‐2 strains would be of great value. We proposed that using a minimal number of crRNAs and Cas13d could target and degrade the SARS‐CoV‐2 and variants in human cells.

CRISPR/Cas13d is an RNA‐targeting CRISPR system with crRNA‐guided Cas13d.^6^ A Cas13d protein and guided RNA were used to cleave the SARS‐CoV‐2 RNA. The crRNAs contain spacer sequences that are complementary to the viral RNA genome specifically (Figure 1). The best advantage of the CRISPR/Cas13d system is that the crRNAs are flexible and easy to adapt to new RNA sequences.^45^ This characteristic enables the rapid design and development of crRNAs to target unexpected virus variants, such as Delta and Omicron, that may escape traditional drugs or vaccines.

Another advantage is the size of Cas13d. The delivery of Cas protein has been of great concern for therapeutic applications. The size of the Cas13d complex is small and it is suitable for adeno‐associated virus (AAV) delivery or lipid nanoparticle (LNP) delivery.^46^ We synthesized 50 crRNAs to target different conserved regions of the SARS‐CoV‐2 virus RNA with high specificity without affecting RNA of the host in human cells. Meanwhile, we have provided a test of the off‐target effect based on the transcriptome of the host cell. We found that the average Q30 level of RNA was more than 85%, and the read length was more than 140 bp, which suggests that no host RNA is degraded. Moreover, we also mapped the genome sequences of coronavirus database though Kraken2, and the results confirmed that almost no coronavirus sequence existed. All these results suggest a high elimination efficiency and low off‐target effect (Table S7). In contrast to previous work, we demonstrated the efficiency and specificity of CRISPR‐Cas13 using authentic SARS‐CoV‐2 infection in human cells.^47^ This is the first study to develop a Cas13d strategy that could target both Delta and Omicron variants.

Therefore, the CRISPR/Cas13d system is a rapid‐to‐develop, mutation flexible, and direct‐degradation approach for the treatment of existing and unexpected SARS‐CoV‐2 variants. In addition to bioinformatic analysis, we also proved the efficacy of this system in eliminating authentic SARS‐CoV‐2 and its VOCs, including Delta and Omicron, in human cells. This novel therapeutic approach not only provides powerful options to fight SARS‐CoV‐2, but also to fight life‐threatening RNA viruses which may evolve and mutate rapidly in the future.

## 4 MATERIAL AND METHODS

### 4.1 Design and cloning of Cas13d‐guided RNAs

For crRNA insertion, we used primers with BbsI restriction sites and annealed the primers to form a double‐stranded DNA for insertion. We used pXR004 (Addgene #109054) and ligated the insert into BbsI‐restricted pXR004. We used the hU6 forward primer in the vector and the specific crRNA reverse primer for colony PCR to verify successful vector construction (Figure 1A,B).

### 4.2 Cloning of the PADM‐CMV‐Cas13d‐FH‐GFP plasmid

The original Cas13d was a gift from Prof. Xiaoxiang Hu lab27. A new Cas13 plasmid was designed by fusing a His‐Flag tag to the Cas13b N‐terminus and GFP to the C‐terminus. pADM‐FH‐GFP and the original Cas13d plasmids were restricted using SgfI/MluI restriction enzymes (1 h, 37°C), followed by purification using NucleoSpin Gel and PCR Clean‐up Kit (Macherey‐Nagel). After digestion and ligation, we collected positive screens using PCR and Sanger sequencing (AGRF, Australia). The plasmid pADM‐CMV‐Cas13d‐mCMV‐GFP was made available through WZ biosciences (pADM‐CMV‐Cas13d‐mCMV‐GFP). The sequences are available in Supporting Information.

### 4.3 Cell culture and transfection

HeLa‐ACE2 cells and Vero‐E6 cells were cultured at 37°C under 5% CO_2_ in DMEM (Gibco) with 10% fetal bovine serum (Gibco), 100 U/ml penicillin, and 100 μg/ml streptomycin. HeLa‐ACE2 cells were transfected using polyethylenimine L (PEI) (Polysciences, 23966‐2, Warrington, PA, USA).

HeLa‐ACE2 cells were co‐transfected with 1 μg of Cas13d expression plasmid and 1 μg different crRNA plasmids for 24 h using PEI 25K (Polysciences, 23966‐1). Details are as follows.

Cells were seeded in 24‐well plates 12–20 h before transfection, and the cell density reached 80% before transfection. The transfection procedure was as follows: two 1.5 ml sterile EP tubes were taken and named tube 1 and tube 2. Fifty microliters of OpTI‐MEM solution was added to the two tubes, plasmid was added to tube 1, and PEI was added to tube 2 (plasmid:PE = 1:3). After standing at room temperature for 5 min, tube 1 solution was slowly added to tube 2 and incubated at room temperature for 10 min. This mixture was slowly added to the cultured cells. After 6–8 h, the medium was changed.

### 4.4 SARS‐CoV‐2 infection and Cas13d transfection

The experiments were performed in a biosafety level‐3 laboratory. SARS‐CoV‐2 (SZTH‐003, D614G, B.1.1.7, B.1.351, and B.1.617) was sourced from COVID‐19 patients. HeLa‐ACE2 cells were incubated with viruses at different MOIs for 2 h at 37°C. Then, the medium was changed, and the cells were incubated with new medium and harvested at indicated time point.

HeLa‐ACE2 cells were transfected with crRNA plasmid (1 μg) and Cas13d (1 μg) in six‐well dishes for 24 h and infected with ancestral or mutant SARS‐CoV‐2 at MOI of 1 for 24 h at 37°C. Cell lysates were analyzed using western blotting.

### 4.5 MOI validation of SARS‐CoV‐2 infection

HeLa‐ACE2 cells were used for infection. A 10‐fold initial dilution of samples was made in octuplicate wells of the 96‐well plates with one freeze–thaw cycle, followed by six serial 10‐fold dilutions. The last row served as a negative control. Four days later, the plates were examined for the appearance of cytopathogenic effect (CPE) under a microscope. Sign of CPE was counted as a positive result. The endpoint titers were calculated with the simplified Reed and Muench method.

### 4.6 Protein extraction and western blotting

The cell lysates were collected and run on the sodium dodecyl sulfate polyacrylamide gel electrophoresis (SDS‐PAGE) and then transferred onto polyvinylidene fluoride (PVDF) membranes (Millipore, IPFL10100). After blocking with bovine serum albumin, primary antibody incubation and secondary antibody incubation, the desired bands were detected using a Super Signal West Pico Chemiluminescent substrate (Bio‐Rad). The following antibodies were used: SARS‐CoV‐2 nucleoprotein (Sino Biological, 40588‐T62), actin (TransGen Biotech, HC201‐02), anti‐mouse IgG (TransGen Biotech, HS201‐01), and anti‐rabbit IgG (TransGen Biotech, HS101‐01).

### 4.7 RNA extraction, cDNA synthesis, and RT‐PCR

First, RNA was extracted. Then, using a standard concentration of RNA, single‐stranded RNA was converted to cDNA using the Revert Aid First Strand cDNA Synthesis Kit (Thermo Scientific, Lithuania, K1622) according to the following PCR program: 42°C for 1 h, 72°C for 5 min, and hold at 4°C. qRT‐PCR was performed with the Bio‐Rad CFX96 using the SYBR Green Supermix (Toyobo, QPK201T) with primers for the indicated genes. FP: 5′‐AACTCTGCCGCTAACGTGAA‐3′, RP: 5′‐AAGCCCAGGTTCTTCTGCTC‐3′; for homosapiens actin NM_001101.3, FP: 5′‐CATGTACGTTGCTATCCAGGC‐3′, RP: 5′‐CTCCTTAATGTCACGCACGAT‐3′; for ORF1as(F1), FP: GAAATTAATACGACTCACTATAGGG, RP: GCGGAGTTGATCACAACTACAGCCATAAC; for N fragment (F2), FP: GAAATTAATACGACTCACTATAGGG, RP: CATTTTGCTCTCAAGCTGGTTCAATCTGTC.

### 4.8 Data analysis

A total of 1,652,991 SARS‐COV‐2 sequence data with high sequencing quality were downloaded from the 2019‐nCoVR (https://ngdc.cncb.ac.cn/ncov/) database on August 1, 2021. The number of mutations in the SARS‐COV‐2 sequence region that paired complementarily with the six crRNA bases was calculated using shell scripts in the Linux environment. The results were visualized using the R‐4.1.1 ggplot2 and ggtree packages. The complete coronavirus sequences were searched using the NCBI nucleotide database. Alignment of coronavirus and crRNA sequences using the local version of BLAST 2.12.0. Mafft‐7.487 was used for multiple sequence alignment, and megaX‐10.2.6 was used to build the phylogenetic tree according to the neighbor‐joining statistical method.

## AUTHOR CONTRIBUTIONS

Z.L., X.G., and C.K. participated in data analysis. L.L., Y.Z., S.Z., and L.Z. participated in cytological experiment. Z.L. and Y.G. participated in manuscript writing. H.Z., M.L., Z.Z., and Y.S. designed the experiment and revised the article. All authors read and approved the final manuscript.

## CONFLICT OF INTEREST

The authors declare they have no conflicts of interest.

## ETHICS STATEMENT

Ethical approval was obtained from the Research Ethics Committee of Shenzhen Third People's Hospital (2021‐030).

## Supporting information

Supporting Information

Supporting Information

Supporting Information

Supporting Information

Supporting Information

Supporting Information

Supporting Information

## Data Availability

The data that support the findings of this study are available from the corresponding author upon reasonable request, and the genome variations of SARS‐CoV‐2 and corresponding metadata were downloaded from China National Center for Bioinformation (CNCB) 2019 Novel Coronavirus Resource (2019nCoVR) database (https://ngdc.cncb.ac.cn/ncov/) on August 1, 2021. The Wuhan‐Hu‐1 (GenBank: MN908947.3) was used as the reference sequence.
